# Membrane Separation Techniques: Advances, Challenges, and Future Avenues

**DOI:** 10.3390/membranes16030112

**Published:** 2026-03-23

**Authors:** Chii-Dong Ho

**Affiliations:** Department of Chemical and Materials Engineering, Tamkang University, Tamsui, New Taipei City 251301, Taiwan; cdho@mail.tku.edu.tw

## 1. Introduction

The separation of substances from one another, such as distillation, extraction, absorption, crystallization, and drying, comprises a main sector of the industry engineering field and has substantial potential to play an increasingly important role in the application of separation technologies and innovations. Membrane separation has become one of the core platform technologies of modern process engineering because it enables modular, often energy-efficient separations across gases, liquids, ions, vapors, organics, and particulates while remaining adaptable to very different industrial settings. Foundational reviews have shown that the field was first developed through strong advances in gas separation and desalination, where polymeric and inorganic membranes were valued for their lower thermal demand, compact design, and scalability, but where performance was consistently constrained by selectivity–permeability tradeoffs, module design, and operating cost considerations. Technological assessments have found wide application at the laboratory scale and beyond to address plant, energy consumption and fouling problems. The nexus between technical feasibility and economic benefits was examined, aiming to create integrated systems at the industrial scale. Baker [1] emphasized that future progress in gas separation would depend not only on better membrane materials but also on realistic process integration, while Bernardo et al. [2] and Ni et al. [3] highlighted how transport selectivity, polymer structure, and mixed-matrix design jointly shape separation performance. In liquid-phase applications, Lee et al. [4], Elimelech and Phillip [5], and Qasim et al. [6] collectively made clear that reverse osmosis and membrane desalination research must be understood not simply as a materials problem, but as a coupled challenge involving energy demand, water quality, system configuration, and environmental constraints. A second major theme in the literature is the persistent importance of fouling, concentration polarization, and wetting. Guo et al. [7], Tijing et al. [8], and Jiang et al. [9] each showed, from different application perspectives, that flux decline and long-term instability remain central bottlenecks, which means that membrane performance cannot be meaningfully evaluated without considering interfacial interactions, hydrodynamics, and cleaning or control strategies. The expected benefits of using emerging separation modules implemented in a porous membrane include improved device performance, and the underlying principles by which the various techniques may be treated are discussed in detail so readers can extrapolate the information to suit their own needs. This same logic is evident in membrane separation, where Osuofa and Husson [10] reviewed how membrane development, configuration design, and process conditions must be considered together. More recent reviews indicate that the field is continuing to broaden in both material scope and methodological sophistication. Winarta et al. [11] documented the rapid rise in metal–organic framework mixed-matrix membranes for gas separation, Hong et al. [12] reviewed MXene-based membranes for ion-selective separations, and the classic polymer–physics framework of Oh et al. [13] remains highly relevant for understanding upper-bound behavior and the structural origins of transport tradeoffs. The artificial intelligence-enabled approaches summarized by Ignacz et al. [14] suggest that membrane science is moving toward a more data-enabled and multiscale phase, in which molecular design, transport modeling, and process optimization are increasingly integrated. The field of membrane science continues to evolve through the combined advancement of membrane materials, transport modeling, module design, hydrodynamics, and application-oriented process engineering. Taken together, these previous studies define the broader context for this Special Issue: membrane research is no longer driven by materials discovery alone, but by the need to connect material architecture, transport physics, fouling control, hydrodynamic design, and application-specific process intensification into robust separation systems.

This Special Issue of *Membranes*, on “Membrane Separation Techniques”, is dedicated to providing a forum that provides comprehensive coverage of the state-of-the-art and the study of advanced applications in separation technology, delivers suitable large-scale design separation processes in various industrial applications, and explores up-to-date techniques and findings. Eleven research articles are included in this Special Issue. The eleven papers gathered in this collection illustrate the breadth of this subject particularly well. They cover pore-scale simulations for membrane distillation, fouling analysis and flux prediction, ultrafiltration hydrodynamics, membrane contactors for CO_2_ capture, analytical microextraction, hybrid liquid membranes, catalytic membrane nanomaterials, metal-ion transport, and nanocomposite membranes for pervaporation. When considered together, these contributions show that membrane research is increasingly shaped by multiscale integration: structure matters, but so do operating conditions, interfacial phenomena, and the ability to translate mechanistic insight into usable engineering design.

## 2. An Overview of Published Articles

The article by Jäger et al. (contribution 1) examined membrane distillation at the pore scale, moving beyond simplified descriptors such as average porosity and tortuosity. The authors reconstructed realistic three-dimensional high-resolution membrane geometries using ptychographic X-ray computed tomography and then simulated multiphase transport with a lattice Boltzmann framework. The phase space is discretized for the LB method, leading to a domain with discrete cubic cells and discrete velocities. They analyzed liquid entry pressure, wetting states on rough hydrophobic surfaces, liquid–membrane contact surfaces, and air–water interfaces inside partially saturated membranes. A key result was that droplet size and distribution within 3D membrane geometries were strongly linked to membrane porosity and local structure. This paper is important because it directly connects real membrane morphology to wetting behavior and transport limitations, offering a more physically grounded basis for the design and optimization of membrane distillation.

The article by Tran et al. (contribution 2) discusses the primary recovery of surfactin from precipitation-treated Bacillus subtilis fermentation liquor through one-stage, dead-end, and cross-flow ultrafiltration (UF). The authors compared membrane materials, molecular-weight cut-offs, and operating modes, while also using Hermia-type modeling to characterize fouling mechanisms via the so-called modified fouling index (MFI) in a specific UF process for effective separation. They further assessed membrane cleaning strategies and found that a 100 kDa polyethersulfone (PES) membrane performed well under cross-flow operation and that the NaOH solution cleaning could effectively regain the permeate flux. This study is valuable because it does not treat separation efficiency and fouling as separate issues; instead, it integrates membrane choice, fouling diagnostics, and cleaning into one practical process framework for biosurfactant recovery.

The article by Chandrasekaram et al. (contribution 3) focused on a dispersive membrane alongside high-performance liquid chromatography (DMME–HPLC) with a sporopollenin–methylimidazolium-based mixed matrix membrane (Sp–MIM-MMM) for measuring substituted phenols in honey, a highly viscous and compositionally complex matrix, offering a more sustainable solution with notable sensitivity and selectivity. The authors used an Sp–MIM-MMM to enrich mono- and disubstituted phenols without derivatization and achieved successful detection in unspiked honey samples. What distinguishes this study is that it goes beyond analytical performance and explicitly evaluates the sustainability of the DMME–HPLC method using the Analytical Eco-Scale (AES) and the Analytical GREEnness Metric (AGREE) approach. As a result, this paper contributes both to membrane-based sample preparation and to the broader agenda of greener analytical chemistry.

Takata and Tanida’s study (contribution 4) investigated how membrane rotation affects ultrafiltration performance for highly concentrated latex emulsions when compared with a conventional cross-flow membrane, since fouling and/or concentration polarization are reduced, clarifying the relationship between the fluid behavior and membrane separation characteristics of a disk-type membrane due to the synergistic effect of particle removal by the centrifugal forces generated by the rotation of the membrane and the reduction in the thickness of the velocity boundary layer. Their experiments showed that rotation-generated shear reduced concentration polarization and particle accumulation near the membrane surface, thereby increasing permeate flux under concentrated conditions. They also demonstrated that shear rate, derived from boundary-layer theory, could unify the interpretation of performance across different membrane diameters and operating conditions. This paper lies translates a device-specific enhancement mechanism into an engineering parameter that is more broadly useful for filtration design with various diameters from the laboratory to the commercial scale.

The article by Ho et al. (contribution 5) examined CO_2_ absorption in a more compact double-unit flat-plate membrane contactors using a monoethanolamine (MEA) solution and embedded three-dimensional turbulence promoters to prevent flow-induced vibration and enhance the CO_2_ absorption rate by overwhelming the concentration’s polarization on the membrane surfaces. A generalized and simplified expression of the average Sherwood number was correlated to calculate the mass transfer coefficient of the CO_2_ absorption in the new design of membrane contactors using turbulence promoter channels. Combining theory and experiment to demonstrate the value and originality of their study, as well as its technical feasibility, the authors showed that properly designed promoters can disrupt boundary layers, mitigate concentration polarization, and increase absorption flux relative to simpler channel configurations. Importantly, the study approached performance improvement from a process-intensification perspective, linking hydrodynamic enhancement to mass-transfer gains rather than relying solely on material substitution. The paper is, therefore, especially relevant to the design and scale-up of gas–liquid membrane contactors.

The article by Ferencz et al. (contribution 6) examined hybrid bulk liquid membranes (BLM) based on dispersion systems, which work by dispersing the aqueous source (SP) and receiving (RP) phases. The authors studied whether transport is possible and under what conditions the system remains operationally stable when the membrane itself is a dispersion of nanoparticles in an organic solvent (NP–OSM). Silver ion (SI) and p-nitrophenol (PNP) were chosen as transportable chemical species, the n-aliphatic alcohols (C6 … C12) as membrane organic solvents, 10–undecenoic acid (UDAc) and 10-undecylenic alcohol (UDAl) as carriers, and magnetic iron oxides as nanoparticles dispersed in the membrane phase with various volumes, membrane thicknesses, temperatures, phase flows, droplet sizes, nanoparticle concentrations, pH gradients, solvent types, salt concentrations, and transported chemical species. The study showed that high separation efficiencies and strong flux enhancement—of almost 10 times for the silver ion and approximately 100 times for p-nitrophenol—were achieved, but only within a limited operating window. Its main contribution is thus to clarify the trade-off between performance and stability, which is critical for the practical use of hybrid liquid membrane systems.

The article by Suárez et al. (contribution 7) developed a mathematical model to predict water-flux decline in direct-contact membrane distillation when inorganic fouling occurs as salts deposited onto the membrane surface, forming an inorganic fouling layer. Their formulation combined steady-state heat and mass transfer with cake-filtration theory to represent the distillate fluxes before and after membrane fouling onset. The model matched experiments well and showed that the onset of membrane failure was relatively constant; the precipitation reaction constant is conditioned by the physicochemical interaction between the feed solution and the membrane; and the rate of flux decline after membrane fouling depends on flow conditions as well as on the precipitation compound. These results indicate that post-fouling flux decline depends not only on precipitate accumulation, but also on hydrodynamic conditions and membrane–solution interactions, suggesting that the cake-filtration theory can be used to represent water flux declines in MD membranes prone to inorganic fouling. The significance of this paper is that it can help to check and replace membranes at optimal times and improve the overall efficiency of this water-treatment system, changing a largely descriptive problem into a predictive one.

The article by Albu et al. (contribution 8) reported the fabrication of catalytic composite membrane nanomaterials by reducing an alcoholic solution of osmium tetroxides (OsO_4_) with high toxicity directly onto polypropylene hollow-fiber supports. An elegant and extremely useful method is presented: the recovery of osmium as a membrane catalytic material in the form of nanoparticles obtained on a polymeric support. The membranes containing osmium nanoparticles (Os–NP) were characterized morphologically and the osmium tetroxide was solubilized in *t*–butanol and the reducing agent, 10–undecenoic acid (UDA), in *i*–propanol, *t*–butanol or *n*–decanol solution. The resulting osmium nanoparticle-containing membrane was structurally characterized via thermal gravimetric analysis using differential scanning calorimetry (TGA, DSC), and through a redox reaction of an organic marker: *p*–nitrophenol (PNP) to *p*–aminophenol (PAP). This study is notable because it turns a hazardous residual chemical into a functional catalytic membrane platform with the technical–economic value of metallic osmium. In doing so, it expands the role of membranes beyond passive separation, moving toward integrated catalytic and reactive applications.

The article by Ho et al. (contribution 9) explored concentric circular membrane contactors fitted with spiral-wired annulus channels for amine-based (monoethanolamide, MEA) CO_2_ absorption under both concurrent- and countercurrent-flow operations. The spiral-wire design generated stronger mixing and reduced concentration boundary-layer thickness, which improved mass transfer and absorption performance. This paper combined theoretical analysis with experimental validation, showing good agreement and obtaining a generalized expression of the average Sherwood number, and also considered the trade-off between absorption flux enhancement and power consumption increment from an economic perspective to assess technical and economic feasibilities. It reinforces the broader lesson that spiral-wired channel design and hydrodynamic control can deliver substantial membrane-contacting gains even when the membrane material itself is unchanged.

The article by Nechifor et al. (contribution 10) studied the transport and recovery of silver ions through *n*–decanol liquid membranes containing 10–undecylenic acid and 10–undecylenyl alcohol carrier molecules in the presence of magnetic oxide nanoparticles through comparison with lead ions, which promote turbulence in the separation module of bulk liquid membranes. By comparing different carriers and optimizing pH, flow conditions, and nanoparticle-assisted agitation, the authors tested for silver ion transport and separation through *n*–decanol liquid membranes with selected carriers by varying the flow of the source and receiving phases, through a pH adjustment of the receiving phase and using a stirring regime with magnetic nanoparticles. The results showed that the process performance (flux and selectivity) substantially improved under the specified hydrodynamic conditions, with the most efficient system being *n*–decanol–10–undecylenic acid–iron oxide nanoparticles, most likely due to the effect of the alkylene group. This paper demonstrates how chemical selectivity and physically induced convective effects can be combined in one liquid membrane system. Its importance lies in showing that transport performance can be tuned simultaneously through carrier chemistry and module-level dynamics.

The article by Allel et al. (contribution 11) produced crosslinked poly(styrene-co-butadiene) (SBR)/Maghnia-organo-montmonrillonite (CSBR/OMMT) nanocomposite membranes to improve the thermal and mechanical properties of the pervaporation separation of methanol/toluene azeotropic mixtures. The thermal properties of these hybrid materials were studied by differential scanning calorimetry and thermogravimetric analysis/thermal differential analysis. The mechanical properties were studied using strength measurements. SBR was prepared by a solvent casting method in situ in the presence of OMMT nanoparticles, through an efficient vulcanization technique using sulfur as a crosslinking agent and zinc diethyldithiocarbamate as a catalyst. Their characterization showed that adding organo-clay improved thermal and mechanical properties, while application tests indicated better total flux and separation performance compared to an appropriate filler-loading range. The study is representative of a mature direction in membrane materials research, in which composition, morphology, robustness, and practical separation behavior are evaluated together rather than in isolation.

## 3. Conclusions

The novel findings and significant results from these eleven contributions reveal the importance of several broad trends in contemporary membrane research. Numerous design schemes in various membrane modules have achieved significant results, allowing for in-depth characterization, with use in practical membrane separation applications. Overall, the eleven papers in this Special Issue introduce guidelines for the sustainable development of these membrane separation processes, pointing to several broad trends in contemporary membrane research. First, there is a clear move toward multiscale understanding, from realistic pore geometry to module-level process design. Second, fouling and concentration polarization remain recurring constraints, but they are increasingly being addressed with predictive modeling and hydrodynamic control. Third, membrane systems are expanding into specialized roles, such as biosurfactant recovery, trace analysis, catalytic transformation, and selective metal transport. Finally, the field is becoming more functionally integrated: membranes are now engineered as platforms that combine selective transport, material functionality, reaction capabilities, and process intensification.
